# SEOM clinical guidelines for diagnosis and treatment of metastatic colorectal cancer 2015

**DOI:** 10.1007/s12094-015-1434-4

**Published:** 2015-12-15

**Authors:** E. Aranda, J. Aparicio, V. Alonso, X. Garcia-Albeniz, P. Garcia-Alfonso, R. Salazar, M. Valladares, R. Vera, J. M. Vieitez, R. Garcia-Carbonero

**Affiliations:** Servicio de Oncología Médica. Instituto Maimónides de Investigación Biomédica (IMIBIC), Hospital Reina Sofía, Universidad de Córdoba, Córdoba, Spain; Red Tematica de Investigación Cooperativa en Cancer (RTICC), ISCiii, Ministerio de Economía y Competitividad, Madrid, Spain; Hospital Universitario y Politécnico La Fe, Valencia, Spain; Hospital Universitario Miguel Servet, Zaragoza, Spain; Harvard T.H. Chan School of Public Health, Boston, USA; Hospital General Universitario Gregorio Marañón, Madrid, Spain; Institut Català d’Oncologia (L’Hospitalet, Barcelona), Barcelona, Spain; Complexo Hospitalario Universitario, A Coruña, Spain; Complejo Hospitalario de Navarra, Pamplona, Spain; Hospital Universitario Central de Asturias, Oviedo, Spain; Hospital Universitario 12 de Octubre, Madrid, Spain

**Keywords:** Colorectal cancer, Metastases, Chemotherapy, Targeted agents, Surgery, Guidelines

## Abstract

Colorectal cancer (CRC) is the second leading cause of cancer dead in Spain. About half the patients will eventually develop distant metastases. However, as treatment options are expanding, prognosis has steadily improved over the last decades. Management of advanced CRC should be discussed within an experienced multidisciplinary team to select the most appropriate systemic treatment (chemotherapy and targeted agents) and to integrate surgical or ablative procedures when indicated. Disease site and extent, resectability, tumor biology and gene mutations, clinical presentation, patient preferences, and comorbidities are key factors to design a customized treatment plan. The aim of these guidelines is to provide synthetic recommendations for managing advanced CRC patients.

## Introduction

In Spain, there were 19,261 new cases of colorectal cancer (CRC) in men (44 cases per 100,000—third most incident cancer site-) and 12,979 cases in women (24.2 cases per 100,000—second most incident cancer site-) in 2012. CRC is the second cause of cancer mortality in men with 8742 deaths (13.7 % of cancer deaths) and in women with 5958 deaths (15.2 % of cancer deaths) annually [1].

The Spanish Society of Medical Oncology (SEOM) invited ten CRC experts based on major scientific contribution in the field. The purpose of this paper was to define current “state of the art” using the methodology of evidence-based medicine. The available medical literature was reviewed according to main topics of disease management, and classified by scientific levels of evidence and grades of clinical recommendation (Table 1) [2]. The resulting text was reviewed, discussed, and approved by all authors.

**Table 1:** Levels of evidence and grades of recommendation [2]

Levels of evidence:
I. Evidence from at least one large randomized, controlled trial of good methodological quality (low potential for bias) or meta-analyses of well-conducted randomized trials without heterogeneity
II. Small randomized trials or large randomized trials with a suspicion of bias (lower methodological quality) or meta-analyses of such trials or of trials with demonstrated heterogeneity
III. Prospective cohort studies
IV. Retrospective cohort studies or case–control studies
V. Studies without control group, case reports, experts opinions
Grades of recommendation
A. Strong evidence for efficacy with a substantial clinical benefit, strongly recommended
B. Strong or moderate evidence for efficacy but with a limited clinical benefit, generally recommended
C. Insufficient evidence for efficacy or benefit does not outweigh the risk or the disadvantages (adverse events, costs,…), optional
D. Moderate evidence against efficacy or for adverse outcome, generally not recommended
E. Strong evidence against efficacy or for adverse outcome, never recommended

## Diagnosis and staging

The extent of the disease must be carefully assessed, as well as tumor biology and patient-related factors before starting cancer-specific therapy. A multidisciplinary team will aim to identify patients with metastatic colorectal cancer (mCRC) for which potentially curative surgical options are appropriate. The suggested procedures are shown in Table 2. The recommended staging system is the 7th edition of the American Join Committee on Cancer’s (AJCC) Cancer Staging [3].

**Table 2:** Suggested staging procedures

History including familial history of tumors and syndromes associated with hereditary disease
Physical examination must include the general condition (performance status, PS), and digital rectal exam
Laboratory tests including liver and renal function and prognostic markers (white blood cell count, alkaline phosphatase, lactate dehydrogenase (LDH), bilirubin, and albumin)
Carcinoembryonic antigen (CEA)
Pathological review of a tumor biopsy should at least provide histological subtype, tumor grade, and *KRAS* and *NRAS* mutational status. *BRAF* genotyping may be considered in *RAS* wild-type tumors for prognostic information
Computed tomography (CT) scan of the chest, abdomen and pelvis. Magnetic resonance imaging (MRI) of the liver could be considered in cases of hepatic metastases
Complete colonoscopy to locate the primary tumor, to obtain tissue for histological diagnosis, and to detect potential synchronous colorectal lesions. Virtual colonoscopy could be useful in case of tumors that impede the progression of the endoscopic tube
Other tests such as a bone scan or a brain CT scan should be performed only if clinically indicated
Additional examinations, as clinically needed, are recommended prior to major abdominal or thoracic surgery with potentially curative intent
Abdominal MRI with intravenous contrast may be considered in patients with potentially resectable liver metastases and for patients with iodine allergy
A fluorodeoxyglucose (FDG)-positron emission tomography (PET–CT) scan should be performed, if available, when metastatic disease is or may potentially become resectable
Needle biopsy of a patient with known histologic diagnosis is only recommended when it may change the therapeutic strategy

## Prognostic classifications

Prognosis of patients treated with modern chemotherapy combinations depends, at a minimum, on clinical characteristics (performance status—PS, comorbid conditions, number, and site of metastases), *BRAF* mutational status [4], and laboratory parameters (Table 2), with a median survival ranging from 14 to 30 months.

The European Society of Medical Oncology (ESMO) proposes assigning patients to one of 4 groups to guide first-line therapeutic strategies (V, C). Group 0 are those patients with liver or lung metastases suitable for potentially curative resection (with clear margins, R0). Group 1 are those patients with limited liver and/or lung metastases that are not R0-resectable upfront but might become resectable after chemotherapy. Patients must be able to undergo major surgery to belong to groups 0 and 1. Group 2 includes those patients with multiple metastasis that present rapid progression and/or tumor-related symptoms and/or risk of rapid deterioration. Patients must be able to tolerate intensive chemotherapy to belong to groups 1 and 2. Those patients that will never have an option for resection, without major symptoms or risk of rapid deterioration or that have severe comorbidity impeding intensive chemotherapy treatment, belong to group 3 [5].

## Biomarkers

Unfortunately, no useful predictive biomarkers have been identified for any chemotherapy or anti-angiogenic drug in mCRC. In contrast, activating mutations in *KRAS* exons 2, 3, and 4 and in *NRAS* exons 2, 3, and 4 have been identified as biomarkers of intrinsic cancer cell resistance to cetuximab or panitumumab [6]. As a result, the European Medicines Agency (EMA) has restricted the use of these drugs to mCRC patients with *KRAS* and *NRAS* wild-type (WT) tumors. No clearly standardized procedures for *KRAS/NRAS* mutational testing have been established and an increasing number of quantitative and highly sensitive techniques are being used [7]. High sensitivity dPCR and NGS platforms are able to pick up circulating tumor *RAS* mutations and other molecular alterations in plasma that drive primary or acquired resistance during anti-epidermal growth factor receptor (EGFR) treatment [8, 9]. All the studies from randomized trials that have validated the predictive value of *RAS* mutations have been performed with available archived paraffin tumor samples, from recent or old primary tumors or metastasis indistinctively, as there is little tumor heterogeneity when evaluating different tumor or metastases locations from the same individuals [10]. More variability has been found in mutation calls from different labs in quality assessment audits [11]. Therefore, the expanded *RAS* mutation analysis needs to be known before anti-EGFR treatment in mCRC, performed on tumor DNA from any location, as long as the performing lab complies with nationally or internationally qualified quality assurance programs (I, A). Plasma can be a surrogate source tissue for mutational analysis when no tumor sample is available or for testing secondary resistance (III, C) (Table 3).

**Table 3:** Summary of recommendations

The European Society of Medical Oncology (ESMO) proposes assigning patients to one of 4 groups to guide first-line therapeutic strategies (V, C)
The expanded *RAS* mutation analysis needs to be known before anti-EGFR treatment in mCRC, performed on tumor DNA from any location, as long as the performing lab complies with nationally or internationally qualified quality assurance programs (I, A)
Plasma can be a surrogate source tissue for mutational analysis when no tumor sample is available or for testing secondary resistance (III, C)
Patients with asymptomatic primary tumor and unresectable disease should start initial palliative chemotherapy. Resection of the primary tumor should only be performed in patients who develop serious complications (II, B)
Surgical R0 resection should be performed for solitary or confined liver or pulmonary metastases (II, A)
CS and HIPEC by experienced expert teams may improve progression-free survival (PFS) and overall survival (OS) for selected patients with PC (IV, B)
For most patients with good PS status and no significant comorbidities, the combination of infused regimens of 5-FU/leucovorin (LV) with either oxaliplatin (FOLFOX) or irinotecan (FOLFIRI) remains the recommended chemotherapy backbone for first-line treatment (I, A)
First-line chemotherapy selection should be based on prior oxaliplatin-based adjuvant treatment, clinical conditions and comorbidities, biologic drug to be combined, and patient’s preferences
Oxaliplatin and capecitabine combination is an alternative first-line treatment option for patients with mCRC (I, B)
In selected patients (i.e., with unresectable, low burden disease, slow tumor growth, mild symptoms, or frailty) a sequential therapy starting with FP or FP plus bevacizumab could be a valid option (I, B) [26–28]
Anti-EGFR antibodies should not be used without prior determination of *RAS* status. Expanded *RAS* analysis is superior to conventional *RAS* analysis (I, A)
Addition of anti-EGFR therapy to FOLFIRI and to FOLFOX improves PFS and OS in first-line treatment of patients with mCRC (II, A)
The addition of bevacizumab to chemotherapy is beneficial with respect to chemotherapy alone (I, B)
There is no clear evidence of the superiority of anti-EGFR over bevacizumab in combination with chemotherapy in the first-line treatment of mCRC
Anti-EGFR agents should not be combined with bevacizumab (I, B)
First-line treatment for fit patients with WT *RAS* mCRC should include a combination of chemotherapy doublet and a monoclonal antibody (anti-EGFR or bevacizumab)
First-line treatment for fit patients with mutant *RAS* mCRC should include a combination of chemotherapy doublet and bevacizumab (I, B)
Second and successive treatment lines should be individualized according to prior therapy, *RAS* status and clinical condition (II, C)
Patients with completely resected metastases should receive perioperatively 6 months of an active, preferably oxaliplatin-based chemotherapy regimen (I, B)
Fit patients with borderline resectable metastases should receive intensive induction therapy with chemotherapy doublets and a monoclonal antibody, or chemotherapy triplets with or without bevacizumab. In *RAS* WT tumors, anti-EGFR may be more effective than bevacizumab in terms of tumor shrinkage (II, B)
Fit patients with technically unresectable metastases and bulky, symptomatic or biologically aggressive disease, should receive intensive first-line therapy with chemotherapy doublets and a monoclonal antibody. In *RAS* WT tumors, bevacizumab may be subjectively better tolerated and allow the patient to receive more lines of therapy. Anti-EGFR agents, however, may be preferred in patients with significant tumor-related symptoms (IV, B)
Treatment de-escalation after induction therapy is often required due to cumulative toxicity, and is also acceptable once disease control is achieved (II, B)
Patients with unresectable metastases who are either unfit or asymptomatic and have limited risk for rapid clinical deterioration, should receive non-intensive/sequential therapy (I, B)

## Role of surgery

Palliative resection for patients with symptomatic primary tumors is mandatory. In patients with an asymptomatic primary tumor and unresectable metastasic disease, primary tumor surgery is controversial. A meta-analysis showed no benefit in survival and quality of life with colectomy in this setting. It is also associated with higher mortality and morbidity rates than in earlier stages, and only 10–20 % patients will present complications requiring surgical treatment [12]. Patients with asymptomatic primary tumor and unresectable disease should start initial palliative chemotherapy. Resection of the primary tumor should only be performed in patients who develop serious complications (II, B).

Surgical resection of colorectal liver metastases (CRLM) is a potentially curative treatment with 5-year survival rates of 20–50 %, but it is only feasible in <15 % of patients. The criteria for resectability of CRLM depend on the experience of the multidisciplinary expert team. Technical aspects like the possibility of all viable tumor to be removed with negative margins while leaving sufficient functional remnant liver (>30 %), and the presence of resectable extrahepatic disease must be considered [13]. Known prognostic factors are laboratory parameters (Table 2), number of metastases, size and location of the lesions, disease-free interval and lymph node stage (for metachronous metastases), tumor grade, and satellite metastases [14]. Resection of lung metastases also offers 25–35 % 5-year survival rates in carefully selected patients. Surgical R0 resection should be performed for solitary or confined liver or pulmonary metastases (II, A).

Peritoneal carcinomatosis (PC) from CRC may occur in up to 50 % of patients. Cytoreductive surgery (CS) with hyperthermic intraperitoneal chemotherapy (HIPEC) consists on performing radical surgery of all visible tumor in the abdomen followed immediately by HIPEC which acts on microscopic residual tumor. One phase III trial and numerous phase II trials with CS + HIPEC suggest improved survival of selected patients with isolated PC from CRC origin, with 5-year survival rates of 30–50 % [15]. CS and HIPEC by experienced expert teams may improve progression-free survival (PFS) and overall survival (OS) for selected patients with PC (IV, B).

## First-line systemic treatment: chemotherapy

Cytotoxic chemotherapy represents the basis for medical treatment of mCRC. Compared to 5-fluorouracil (5-FU) alone, combinations are more effective but also more toxic [16, 17]. Irinotecan is associated with neutropenia, alopecia, and gastrointestinal side effects (nausea, vomiting, diarrhea, and mucositis). Oxaliplatin-based combinations are associated with neutropenia, thrombocytopenia, diarrhea and sensory neuropathy, and the main dose-limiting toxicity. Infusional 5-FU schedules are less toxic than bolus regimens and should preferably be used. Combinations of bolus 5-FU with either irinotecan or oxaliplatin are not recommended based on higher toxicity and poor outcomes [18]. For most patients with good PS status and no significant comorbidities, the combination of infused regimens of 5-FU/leucovorin (LV) with either oxaliplatin (FOLFOX) or irinotecan (FOLFIRI) remains the recommended chemotherapy backbone for first-line treatment (I, A).

Several direct comparisons of the addition of oxaliplatin versus irinotecan to a LV/5-FU regimen did not show any difference in first-line therapy in terms of response rate (RR) and PFS [19, 20]. Thus, first-line chemotherapy selection should be based on prior oxaliplatin-based adjuvant treatment, clinical conditions and comorbidities, biologic drug to be combined, and patient’s preferences.

Capecitabine is an oral fluoropyrimidine (FP) with similar efficacy to bolus 5-FU/LV in the first-line treatment of mCRC [21]. The most common adverse events are gastrointestinal (diarrhea, nausea, vomiting, and stomatitis) and hand-foot syndrome. Capecitabine in combination with oxaliplatin is considered to have an efficacy (PFS and OS) similar to that of FOLFOX [22]. Thus, the oxaliplatin and capecitabine combination is an alternative first-line treatment option for patients with mCRC (I, B). Toxicity with capecitabine and irinotecan combinations is higher, mainly gastrointestinal.

Retrospective analysis indicates that the use of all three cytotoxics (FP, oxaliplatin, and irinotecan) in various sequences may result in the longest survival [23]. However, some evidences suggest that initial polychemotherapy is not essential in all cases [24, 25]. In selected patients (i.e., with unresectable, low burden disease, slow tumor growth, mild symptoms, or frailty) a sequential therapy starting with FP or FP plus bevacizumab could be a valid option (I, B) [26–28].

One randomized phase III trial showed increased RR, resectability of metastases ,and survival with the FOLFOXIRI combination, at the expense of higher toxicity [29]. The combination of bevacizumab and FOLFOXIRI has shown to significantly improve RR and PFS in selected mCRC patients when compared to FOLFIRI plus bevacizumab, again with increased incidence of adverse events [30].

## First-line systemic treatment: targeted therapies

First-line targeted therapies include the anti-vascular endothelial growth factor (VEGF) agent bevacizumab and the anti-EGFR agents, cetuximab and panitumumab. *RAS* status is the main factor involved in the decision about anti-VEGF or anti-EGFR strategy [31, 32]. Anti-EGFR antibodies should not be used without prior determination of *RAS* status. Expanded *RAS* analysis is superior to conventional *RAS* analysis (I, A).

### Management of patients with wild-type (WT) mCRC after expanded RAS analysis

The CRYSTAL trial compared FOLFIRI versus FOLFIRI with cetuximab in first-line mCRC. The cetuximab-containing arm provided benefits in OS, PFS, and RR in *RAS* WT patients [31]. The PRIME trial compared the combination of FOLFOX4 versus FOLFOX4 + panitumumab in previously untreated mCRC. In the *RAS* expanded analysis, PFS and OS were more favorable for the combination group [32]. Some studies suggest that antiEGFR agents should not be combined with oxaliplatin-based, non-infusional FP schedules. Addition of anti-EGFR therapy to FOLFIRI and to FOLFOX improves PFS and OS in first-line treatment of patients with mCRC (II, A). Randomized trials have also demonstrated improved PFS and/or OS when bevacizumab is added to irinotecan-, oxaliplatin-, and FP-based chemotherapy [26–28, 33, 34]. The addition of bevacizumab to chemotherapy is beneficial with respect to chemotherapy alone (I, B). After analysis of expanded *RAS* mutations, RR and median OS were better for FOLFIRI plus cetuximab compared to FOLFIRI plus bevacizumab in the FIRE-3 trial [35]. Schwartzberg et al. [36] compared mFOLFOX6 plus panitumumab or bevacizumab in a randomized phase II trial. In the *RAS* WT subgroup, PFS and median OS were greater for the panitumumab arm. The phase III CALGB/SWOG 80405 study evaluated the combination of FOLFIRI or mFOLFOX6 with bevacizumab or cetuximab [37]. After a preliminary analysis of expanded *RAS* mutations, no differences in median PFS and OS have been found. A meta-analysis of the three studies showed an increase in RR and OS with first-line anti-EGFR therapy compared with anti-VEGF therapy in *RAS* WT mCRC [38]. There is no clear evidence of the superiority of anti-EGFR over bevacizumab in combination with chemotherapy in the first-line treatment of mCRC. Two randomized trials have demonstrated that combination of chemotherapy with both anti-EGFR and bevacizumab is deleterious [39, 40]. Anti-EGFR agents should not be combined with bevacizumab (I, B). First-line treatment for fit patients with WT *RAS* mCRC should include a combination of chemotherapy doublet and a monoclonal antibody (anti-EGFR or bevacizumab).

### Management of patients with mutated mCRC after expanded RAS analysis

As aforementioned, the addition of bevacizumab to irinotecan-, oxaliplatin-, or FP-based chemotherapy is beneficial in comparison with the administration of chemotherapy alone, independently of the *RAS* status. However, the addition of anti-EGFR therapies has a potential detrimental effect in *RAS*-mutated patients. First-line treatment for fit patients with mutant *RAS* mCRC should include a combination of chemotherapy doublet and bevacizumab (I, B).

## Second- and successive treatment lines

**Fig. 1:**
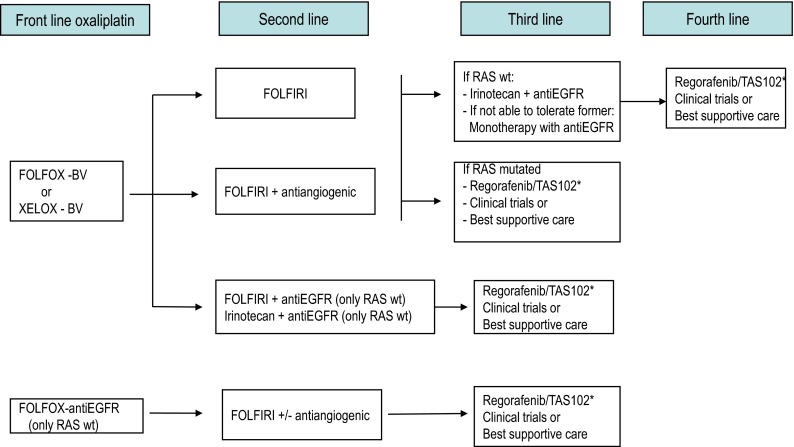
Therapeutic strategies in advanced colorectal cancer. Patients appropriate for intensive therapy. *Note* Front line treatment should consider clinical symptoms, comorbid conditions, prior adjuvant therapy, tumor biology and dynamics, and potential ability for metastasis resection. *BV* bevacizumab, *XELOX* oxaliplatin + capecitabine, *FOLFOX* biweekly oxaliplatin + infusional 5FU/LV, *FOLFIRI* biweekly irinotecan + infusional 5FU/LV, *wt* wild type. *If available

**Fig. 2:**
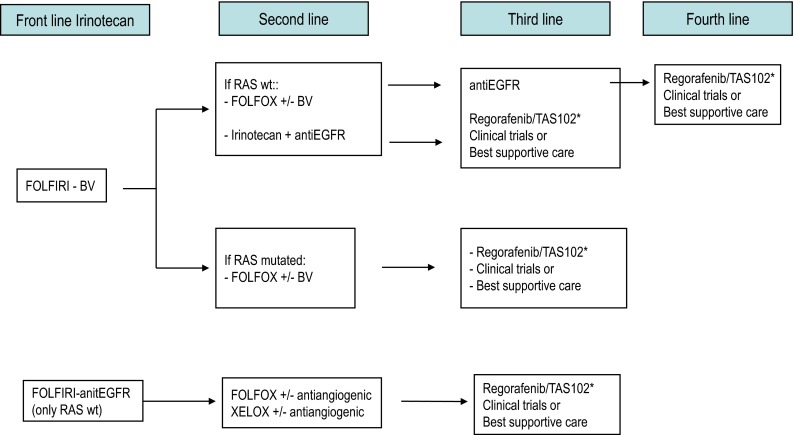
Therapeutic strategies in advanced colorectal cancer. Patients appropriate for intensive therapy. *Note* Front line treatment should consider clinical symptoms, comorbid conditions, prior adjuvant therapy, tumor biology and dynamics, and potential ability for metastasis resection. *BV* bevacizumab, *XELOX* oxaliplatin + capecitabine, *FOLFOX* biweekly oxaliplatin + infusional 5FU/LV, *FOLFIRI* biweekly irinotecan + infusional 5FU/LV, *wt* wild type. *If available

**Fig. 3:**
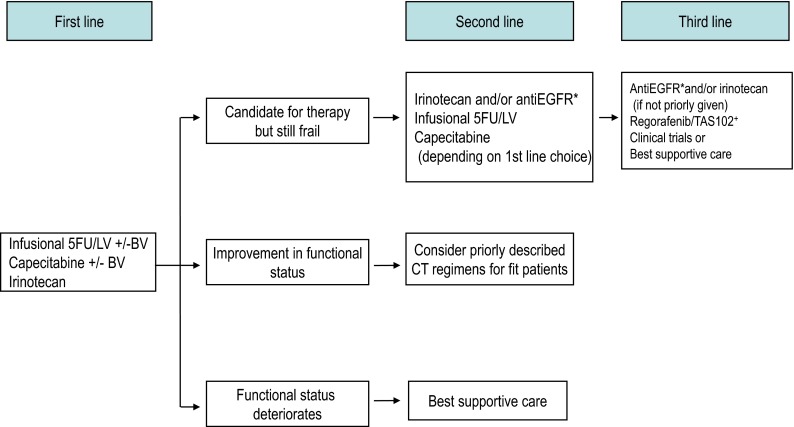
Therapeutic strategies in advanced colorectal cancer in patients who cannot tolerate intensive therapy. *BV* bevacizumab, *5FU/LV* 5-fluorouracil/leucovorin, *CT* chemotherapy. *RAS wild type only. ^+^If available

Therapy after first progression will depend on prior treatments (Figs. 1, 2, 3). For patients who received oxaliplatin-based therapy, FOLFIRI, or irinotecan alone are the preferred options. When the previous treatment was an irinotecan-based combination, the recommended options are FOLFOX or XELOX. With respect to the use of targeted therapies, available options are as follows:

- For patients treated with first-line bevacizumab-containing chemotherapy, the continuation of bevacizumab in conjunction with a second-line chemotherapy improves OS [41] and PFS [41, 42] as compared to just switching the chemotherapy regimen alone.
- It may also be appropriate adding bevacizumab to chemotherapy if it was not used in initial therapy, preferably in combination with oxaliplatin-based therapy [43].
- For patients previously treated with oxaliplatin-based therapy, FOLFIRI plus aflibercept is an option, particularly if they did not receive prior bevacizumab therapy [44].
- Other alternative after progression to FOLFOX plus bevacizumab in first line is FOLFIRI plus ramucirumab [45].
- For patients with expanded *RAS* WT mCRC, cetuximab, or panitumumab plus preferably irinotecan-based therapy [46, 47] is recommended in second-line treatment. They may also be employed as single agents in third or subsequent lines of therapy in patients naive of anti-EGFR therapy [48, 49]. Cetuximab and panitumumab appear to have comparable efficacy when used as single agents for salvage therapy in patients with chemotherapy-refractory mCRC [50]. The reintroduction of EGFR inhibitors in subsequent treatment lines is not recommended for previously exposed patients.

Regorafenib [51] and TAS 102 [52] may be considered for patients who have progressed to all three chemotherapeutic drugs, bevacizumab and anti-EGFR agents, that still conserve adequate PS and organ function.

Second and successive treatment lines should be individualized according to prior therapy, *RAS* status, and clinical condition (II, C).

## Treatment strategy

As treatment options continue to expand for mCRC, selection of the most appropriate therapeutic regimen and administration sequence, as well as their integration with other treatment modalities (i.e., surgery or ablative therapies), is becoming increasingly complex. Since the choice of first-line treatment will compromise subsequent treatment options, it is important to plan upfront a temptative therapeutic strategy, particularly in those patients with unresectable disease, within the concept of the continuum of care. Some practical recommendations to tailor the therapeutic strategy according to the four clinically defined groups by ESMO are provided below [5]. Suggested treatment sequence is shown in Figs. 1, 2, and 3 [53].

### Group 0

In patients with resectable metachronous metastasis, perioperative chemotherapy with FOLFOX has shown to modestly improve disease-free survival (DFS) with a non-significant trend towards improved survival. Initial resection of metastases followed by adjuvant chemotherapy is an alternative option. In patients with resectable synchronous metastases, integration of the surgical strategy with perioperative chemotherapy shall be carefully customized in each patient considering location of primary tumor and metastasis, their size and extent, local symptoms, patient’s comorbid conditions, and the expected liver remnant after resection. Preoperative chemotherapy followed by synchronous or staged colectomy and liver or lung resection may be considered. Colectomy may be followed by neoadjuvant chemotherapy and a staged resection of metastatic disease, or may be performed with synchronous or subsequent liver or lung resection, followed by post-operative chemotherapy. Patients with completely resected metastases should receive perioperatively 6 months of an active, preferably oxaliplatin-based chemotherapy regimen (I, B).

Ablative therapy of liver metastases using radiofrequency, cryosurgery, or external radiotherapy (radiosurgery, SBRT, and IMRT) is an alternative strategy if surgical resection is not technically feasible or medically advisable [54].

### Group 1

In patients with initially unresectable, organ-confined metastases, systemic chemotherapy may induce sufficient cytoreduction to enable subsequent resection. The achievement of a disease-free status is a highly desirable goal and the only means for the patient to potentially achieve long-term survival. With this aim, the most active induction regimens shall be administered upfront in patients able to tolerate it, generally chemo-doublets combined with a monoclonal antibody or chemo-triplets. Different combinations of either oxaliplatin or irinotecan with FP are considered suitable chemotherapy options with similar efficacy but different toxicity profiles. Cross-trial comparisons and prospectively planned subgroup analysis from the FIRE-3 trial suggest that anti-EGFR agents may be more effective in terms of tumor shrinkage than bevacizumab combinations [35–38]. Triplet combination with FOLFOXIRI, with or without bevacizumab, may be also considered in selected patients [29, 30]. Anyway, potential conversion to resectability has to be reevaluated every 2 months and surgery scheduled as soon as possible, to minimize chemotherapy-induced liver toxicities and perioperative morbidity. Fit patients with borderline resectable metastases should receive intensive induction therapy with chemotherapy doublets and a monoclonal antibody, or chemotherapy triplets with or without bevacizumab. In *RAS* WT tumors, anti-EGFR may be more effective than bevacizumab in terms of tumor shrinkage (II, B).

### Group 2

In patients with technically unresectable metastasis but adequate PS, and bulky, symptomatic or biologically aggressive disease, intensive first-line therapy is advised aiming to induce early tumor regression/control and/or symptomatic relief. A chemotherapy doublet in combination with a targeted agent is generally recommended for these patients (Figs. 1, 2). Upfront bevacizumab-based therapy would provide patients more treatment options in the long run, allowing EGFR-targeted agents to be used later in the course of the disease. However, first-line therapy with anti-EGFR monoclonal antibodies is also a reasonable option in patients with *RAS* WT tumors, particularly in those with significant tumor-related symptoms due to the earlier onset of response induced by these agents. Fit patients with technically unresectable metastases and bulky, symptomatic, or biologically aggressive disease, should receive intensive first-line therapy with chemotherapy doublets and a monoclonal antibody. In *RAS* WT tumors, bevacizumab may be subjectively better tolerated and allow the patient to receive more lines of therapy. Anti-EGFR agents, however, may be preferred in patients with significant tumor-related symptoms (IV, B).

Maintenance treatment with FP and bevacizumab has been shown to prolong PFS with no impact on OS as compared to complete discontinuation of therapy. Treatment de-escalation after induction therapy is often required due to cumulative toxicity, and is also acceptable once disease control is achieved (II, B).

### Group 3

Patients with definitively unresectable disease, unfit due to comorbid conditions, and/or with no present or imminent symptoms and limited risk for rapid deterioration, are good candidates for non-intensive therapy. The therapeutic aim in these patients is to slow tumor progression and improve life expectancy with minimum treatment burden. Initial FP monotherapy is a common upfront treatment option in these patients, with or without bevacizumab (Fig. 3). Irinotecan or raltitrexed may also be suitable options for patients in whom FP are not warranted (i.e., severe vascular disease, DPD deficient). If functional status improves following therapy, more intensive therapeutic strategies may be considered. If health status deteriorates, best supportive care is the recommended option. If the patient is still frail but candidate for further therapy upon disease progression to FP, irinotecan may be administered, either alone or, in *RAS* WT tumors, in combination with EGFR-targeted agents. Cetuximab or panitumumab may be also employed as single agents in patients with progressive disease after prior irinotecan-based treatment. Patients with unresectable metastases who are either unfit or asymptomatic and have limited risk for rapid clinical deterioration, should receive non-intensive/sequential therapy (I, B).
